# Green Chemistry Based Synthesis of Zinc Oxide Nanoparticles Using Plant Derivatives of *Calotropis gigantea* (Giant Milkweed) and Its Biological Applications against Various Bacterial and Fungal Pathogens

**DOI:** 10.3390/microorganisms10112195

**Published:** 2022-11-04

**Authors:** Ammara Farooq, Umair A. Khan, Haider Ali, Manda Sathish, Syed Atif Hasan Naqvi, Shehzad Iqbal, Haider Ali, Iqra Mubeen, Muhammad Bilal Amir, Walid F. A. Mosa, Alaa Baazeem, Mahmoud Moustafa, Sulaiman Alrumman, Ali Shati, Sally Negm

**Affiliations:** 1 The Institute of Molecular Biology and Biotechnology, The University of Lahore, Sargodha Campus, Sargodha 40100, Pakistan; 2 School of Bioscience, University of Birmingham, Birmingham B15 2TT, UK; 3 Centro de Investigación de Estudios Avanzados del Maule (CIEAM), Vicerrectoría de Investigación y Postgrado, Universidad Católica del Maule, Talca 3460000, Chile; 4 Department of Plant Pathology, Faculty of Agricultural Sciences and Technology, Bahauddin Zakariya University, Multan 60800, Pakistan; 5 College of Plant Science and Technology, Huazhong Agricultural University, Wuhan 430070, China; 6 Department of Entomology, Faculty of Agricultural Sciences and Technology, Bahauddin Zakariya University, Multan 60800, Pakistan; 7 State Key Laboratory of Rice Biology, Ministry of Agriculture, Key Laboratory of Molecular Biology of Crop Pathogens and Insects, and Key Laboratory of Biology of Crop Pathogens and Insects of Zhejiang Province, Institute of Biotechnology, Zhejiang University, Hangzhou 310058, China; 8 South China Botanical Garden, Chinese Academy of Sciences, Guangzhou 510650, China; 9 College of Life Sciences, Gannan Normal University, Ganzhou 341000, China; 10 Plant Production Department (Horticulture-Pomology), Faculty of Agriculture, Alexandria University, Alexandria 21531, Egypt; 11 Department of Biology, College of Science, Taif University, Taif 21944, Saudi Arabia; 12 Department of Biology, Faculty of Science, King Khalid University, Abha 62529, Saudi Arabia; 13 Department of Botany and Microbiology, Faculty of Science, South Valley University, Qena 83523, Egypt; 14 Department of Life Sciences, College of Science and Art Mahyel Aseer, King Khalid University, Abha 62529, Saudi Arabia; 15 Unit of Food Bacteriology, Central Laboratory of Food Hygiene, Ministry of Health, Branch in Zagazig, Zagazig 44511, Egypt

**Keywords:** nanotechnology, intelligent agriculture, green synthesis, *Calotropis gigantea*, Ak-plant

## Abstract

Nanotechnology is a burning field of scientific interest for researchers in current era. Diverse plant materials are considered as potential tool in green chemistry based technologies for the synthesis of metal nanoparticles (NPs) to cope with the hazardous effects of synthetic chemicals, leading to severe abiotic climate change issues in today’s agriculture. This study aimed to determine the synthesis and characterization of metal-based nanoparticles using extracts of the selected plant *Calotropis gigantea* and to evaluate the enzyme-inhibition activities and antibacterial and antifungal activity of extracts of metal-based zinc nanoparticles using *C. gigantea* extracts. The crystal structure and surface morphology were characterized by X-ray diffraction (XRD) and scanning electron microscopy (SEM). *C. gigantea* was examined for antimicrobial activity against clinical isolates of bacteria and fungi. The water, ethanolic, and acetone extracts of *C. gigantea* were studied for their antagonistic action against bacterial strains (*E. coli*, *S. aureus*, *P. multocida*, and *B. subtilis*) and selected fungal strains (*A. paracistic*, *F. solani*, *A. niger*, *S. ferrugenium,* and *R. nigricans*). In vitro antimicrobial activity was determined by the disc diffusion method, where *C. gigantea* was tested for *AChE* and *BChE* inhibitory activity using Ellman’s methodology. The kinetic analysis was performed by the proverbial Berthelot reaction for urease inhibition. The results showed that out of all the extracts tested, ethanolic and water extracts possessed zinc nanoparticles. These extracts showed the maximum zone of inhibition against *F. solani* and *P. multocida* and the lowest against *S. ferrugenium* and *B. subtilis*. A potential source of *AChE* inhibitors is certainly provided by the abundance of plants in nature. Numerous phyto-constituents, such as *AChE* and *BChE* inhibitors, have been reported in this communication. Water extract was active and has the potential for in vitro *AChE* and *BChE* inhibitory activity. The urease inhibition with flower extracts of *C. gigantea* revealed zinc nanoparticles in water extracts that competitively inhibited urease enzymes. In the case of cholinesterase enzymes, it was inferred that the water extract and zinc nanoparticles have more potential for inhibition of *BChE* than *AChE* and urease inhibition. Furthermore, zinc nanoparticles with water extract are active inthe inhibition of the bacterial strains *E. coli*, *S. aureus*, and *P. multocida* and the fungal strains *A. paracistic*, *F. solani*, and *A. niger*.

## 1. Introduction

Nanotechnology has become a very revolutionary and dynamic area of research at the interface between chemical engineering, materials science, biotechnology, and nanotechnology [1]. It is a field of science and technology about the control of matter anatomic and molecular scales [2,3]. Nanosized materials have led to the massive-scale production of nanoparticle-containing products and NPs [4]. These NPs are identified as particles less than 100 nm in size [5]. The physicochemical and biological properties of nanomaterials are very distinct. These particles have significantly greater volume-to-mass, the surface area that raises chemical reactions and biological activity than biomolecules but are of the same scale [6].

Nanomaterials are used in many industrial applications and goods, including sunscreens and cosmetics, and for a broad range of medical uses, such as experimental drug delivery and imaging [7]. Nanomaterial-like metal NPs are made from metal precursors [8], and in existing use, they are metal-based NPs, viz., zinc oxide, titanium dioxide, iron oxide, and nano silver. Nanoparticles equipped with drugs may address the shortcomings of toxicity, insufficient transmission, and enzyme depletion compared with standard antimicrobial agents. These are eco-friendly and biological methods of synthesis with microbial enzymes [9], fungi [10], and plants or plant extracts. This biological synthesis of nanoparticles is known as the green synthesis of nanoparticles [11]. Green chemistry is a modern approach to pollution control once environmental issues emerge [12]. Green chemistry has the potential to create the cheapest and least harmful energy-intensive complementary approaches to the synthesis of nanoparticles seen in recent decades [13].

Plants are excellent metallic nanoparticle synthesizers [14]. The use of plant extracts as a substitute for nanoparticle production has also been preferred in this aspect [15]. The extraction might be made from various sections of the plants including stems, vines, leaves, roots, shoots, petioles, seeds, and peel. Furthermore, the plant-mediated green chemistry route using a plant with sufficient phytochemicals was reported to be quicker, more durable, and more affordable [16].

In the pharmaceutical and biopharmaceutical fields, there are many remarkable applications of plant-synthesized and metal NPs [17]. Nanoparticles, along with plant extracts, have significant potential for a wide range of biological applications, such as antibacterial and anti-inflammatory agents for antibiotic-resistant bacteria, preventing infections and healing wounds [18]. Plants’ medicinal advantages lie in certain chemical compounds that generate specific effects on the human body, such as antibiotics and enzyme inhibitors. Alkaloid tannins, flavonoids, and phenolic compounds are the most vital biologically active plant compounds that act as antimicrobials [19]. Due to the nontoxic, safe, inorganic, and antibacterial nature of these agents known as nanoparticles, they have been used for centuries and are capable of killing approximately 650 microorganisms that cause diseases [20]. Furthermore, enzyme inhibition activities are performed by using biologically synthesized metal nanoparticles to cure diseases caused by the excessive release of enzymes in the body [21]. Enzyme activities are related to catalytic activity [22]. Enzymes such as urease are metalloenzymes of nickel derived from plants. Urease in plants is a homo-oligomeric enzyme [23]. Hydroxamic acid, imidazole derivatives, and bismuth complexes are widely available urease blockers [24]. Prior studies on biologically synthesized nanoparticles of bioactive metal oxides showed remarkable therapeutic potential [25].

*Calotropis gigantea* (L.) belongs to the Asclepiadaceae family, which has gained popularity owing to its large use in conventional medicine as an enzyme inhibitor and antimicrobial [26,27,28]. *Calotropis gigantea* is also a xerophytic shrub that is erect. The plant is renownedfor producing large volumes of latex constraints [29]. *Calotropis gigantea* is used to combat weakness, swelling, and persistent fevers [30]. Its flowers have been used to treat hypertension, catarrh, anorexia, helminthic diseases, allergy, asthma, and fever conditions. The plant’s root bark is often used in dermal diseases, gut worms, helminthic infections, coughing, and ascites [31]. The plant roots in powdered form treat asthma, bronchitis, and dyspepsia, and promote stomach secretions [28].

This research was designed to develop effective and eco-friendly management approaches for disease-causing bacteria and fungi. In this research, the efficacy of green-synthesized zinc oxide NPs along with *C. gigantean* leaf, flower, and stem extracts using different solvents (ethanol, water, and acetone) was investigated. Biological activities, such as antimicrobial, antifungal, and enzyme inhibition activities, were also examined using characterization techniques. This technique successfully provided the best management tactics against fungi and bacteria in the field.

## 2. Materials and Methods

### 2.1. Plant Material

Fresh flowers, stems, and leaves of *C. gigantean* were harvested from the surroundings of the satellite town of Sargodha, Punjab.

### 2.2. Collection of Culture Media of Various Fungi and Bacteria

Four bacterial and five fungal strains, viz., *E. coli*, *S. aureus*, *P. multicide*, *B. subtilis*, *Aspergillus parasiticus*, *Fusarium solani*, *Aspergillus niger*, *S. ferrugenium,* and *Rhizopus nigaricans* were collected from the University of Lahore (Sargodha campus, Punjab).

### 2.3. Preparation of Plant Extracts

Fresh leaves, stems, and flowers of *C. gigantea* were cleaned with water to eliminate any contamination, dried at ambient room temperature, and crushed to a powdered form. The powder of leaves was divided into three portions and dissolved in acetone, methanol, or distilled water. They were plunged into sealed plastic bottles, decanted, and the resultant contamination was cleaned off. Fresh plant parts were sliced into tiny parts, and 10 g was added to a 100 mL distilled water beaker where the mixture was heated at 60 °C for 45 min with occasional stirring and permitted to chill at room temperature. Acetone and methanol extracts were evaporated by leaving the bottle lid off. Plant extracts were weighed and mixed in 10% dimethyl sulfoxide (DMSO) to create a stock solution through which low doses were formulated.

### 2.4. Green Synthesis of Nanoparticles

All prepared reagents were efficient for research without further filtration. Zinc acetate Zn (C_4_H_6_O_4_) was purchased from Sigma–Aldrich with a purity of approximately 99.5%. Triethylene glycol (TREG) was added as a polyol solvent that had the ability to dissolve zinc acetate and abundant –OH groups, which serve as hydrolysis agents for zinc acetate. Fresh powder of leaves, flowers, and stems of *C. gigantea* was obtained, and distilled water was used toform water solutions. The powder of zinc acetate was soaked in deionized water to make a 10 mM zinc acetate standard solution through which a sequence of solutions of 1–5 mM wasproduced. Zinc acetate solution was blended with water extract of *C. gigantea* fresh leaves. The extract of flowers and stems wasplaced in a flask at a ratio of 1.1 (*v*/*v*) to prepare a 50 mL solution. The flask was covered with an aluminum sheet and then warmed for 5 h in a water bath at 60 °C. To check antimicrobial activity, the mixture was stored in afreezer and then further examined using a UV–Vis spectrophotometer and SEM.

### 2.5. Characterization of Zinc Nanoparticles

#### 2.5.1. Ultraviolet Spectroscopy (UV-Vis)

Ultraviolet-Vis spectroscopy analysis was used to confirm ZnO NP synthesis based on their optical properties. Ultraviolet (Manimegalai et al., 2003) visible spectroscopic analysis was performed with the help of a spectrometer (Shimadzu 1800 Japan). The UV–visible spectrophotometer occurred in the wavelength range of 200–600 nm.

#### 2.5.2. SEM Analysis

The structure and composition of freeze-dried purified ZnO NPs wereanalyzed by using a 10 kV ultra-high-resolution scanning electron microscope with 25 µL of the sample with sputter coated on a copper stub, and the images of ZnO NPs were studied using an FEI QUANTA-200 SEM. The image indicated that nanoparticles were well distributed with the lowest agglomeration of nanoparticles. 

#### 2.5.3. EDX Analysis

For energy dispersive X-ray (EDX) analysis, the particles were dried on a carbon-coated copper grid and the analysis was performed on the SEM instrument equipped with aThermoEDX attachment. The EDX analysis spectrum indicated the formation of ZnO NPs.

#### 2.5.4. FTIR Analysis

The FTIR analysis of the zinc oxide nanoparticles was performed by a Perkin Elmer brandModel Spectrum RX1 (Range 400–4000 cm^−1^). 

### 2.6. Enzyme Inhibition Assays

#### 2.6.1. Inhibition of ACHE and BCHE

The inhibition assay was carried out using the Ellman technique of acetylcholinesterase (AChE) and butyl-cholinesterase (BChE) (Ellman et al., 1961). AChE, BChE, and DTNB display a colored complex named 5-thio-2-nitrobenzoate. The concentration was determined utilizing a μQuant microplate spectrophotometer by enhancing the absorbance at 412 nm. A few other reagent kitswere used, such as acetyl-thiocholine iodide and butyryl-thiocholine iodide. Donepezil and galantamine were identified as control drugs. Phosphate-buffered saline (0.1 M (_KH2PO4/K2HPO4_) at pH 8.0, a stock solution of synthesized metal compounds was made. A controlled volume of DTNB (the reagent for Ellman) and plant extracts (0.03 U/mL) containing enzymes (AChE and BChE) were prepared to interact for 10 min by pre-incubation at 30 °C. The plant extracts had an amount of 0.03 U/mL(AChE and BChE) and were prepared to respond at 30 °C for 15 min at pre-incubation. After the application of 1 mM ATCI or BTCI, each reading was taken three times after incubation for 15 min, and the IC_50_ values of the substances were determined by plotting the sample concentration against the inhibition, whereas donepezil and galantamine were used as standard inhibitors for the experiment.

#### 2.6.2. Inhibition of Urease

The enzyme assay [32] is an improved version of the widely recognized Berthelot reaction. A cumulative concentration of 85 μL of different extractscomprised 10 μL of pH 7.0 phosphate buffer solution in the well plate with 96 wells along with 10 μL sample extract, and 25 μL enzyme solution (0.1347 units) was added to this. The product was blended and incubated at 37 °C for 5 min. Then, 40 microlitres of urea stock solution (20 micromolar) was added to each microwell, and incubation was started for another 10 min at 37 °C. Then, 115 μL phenol hypochlorite reagent was added to each well after a specified period (prepared by combining 45 μL phenol reagent with 70 μL alkali reagent). Incubation was performed at 37 °C again for the next 10 min for color improvement. The absorption spectrum was calculated at 625 nm wavelength using the Synergy HT 96-well plate test. Thiourea was used as the standard inhibitor, while the percent inhibition of enzymes was measured by using the following formula:

(1) $$Inhibition\;\left(\%\right)=100-\frac{Absorbance\;of\;test\;sample}{Absorbance\;of\;control}\times 100$$

IC_50_ values (which means that the enzyme-catalyzed mechanism occurs at a strength of 50 percent) of extracts were identified using EZ-Fit Enzyme Kinetics Software.

### 2.7. Antimicrobial Activity of Plant Extracts and Zn Nanoparticles

The antimicrobial activities of plant extracts and nanoparticles were investigated against *E. coli*, *S. aureus*, *P. multocida*, *B. subtilis*, and fungal strains such as *Aspergillus parasiticus*, *Fusarium solani*, *Aspergillus niger*, *S. ferrugenium*, and *Rhizopus nigarican*. Both laboratory appliances and growing media were disinfected for 30 min at 115 °C and 15 psi by autoclaving. The disc diffusion approach was used to examine antimicrobial activity. A stock of bacteria was prepared to replicate and rejuvenate bacteria by inoculation through culture media of *E. coli*, *S. aureus*, *P. multocida*, *B. subtilis*, *Aspergillus parasiticus*, *Fusarium solani*, *Aspergillus niger*, *S. fermentium,* and *Rhizopus nigarican* in 5 mL of nutrient agar solution and then implanted in an incubator at 37 °C for 24 h. Preparation of research bacteria was performed by injecting one cultivated bacterial inoculation loop into a 5 mL NaCl solution of 0.19%. In addition, it was diluted, and its fecundity was modified to 0.5 in the McFarland turbidity approach of 180 colonies forming units/mL by adding artificially grown media. The method of inhibition was used to test the antibacterial activity. A petri dish was filled with 20 mL of nutrient agar mixture and disinfected for fifteen minutes until the solution was firmed in nutrient agar. Then, 0.1 mL of the microbial solution was added to the growing solution of the nutrient broth culture of the standard drug (Rifampicine) for antibacterial function control for antifungal infection (Fluconazole). Then, it was incubated at 37 °C for 24 h before being used to calculate the clear zone diameter.

#### 2.7.1. Disc Diffusion Method

The disc diffusion system was used to check the antibacterial action of the plant *C. gigantea* and its sections, such as its fruit, leaves, and roots. First, methanol acetone was dissolved in DMSO at 100 mg/mL, after whichwater, methanol, and acetone extracts of the stem, flower, and leaves were pre-filtered through 0.45 μm sterilization filter layers ofbacteria takenata concentration of 100 µL, and 108 fungal inoculum colonies forming units/mL were distributed onto culture plates. Mueller–Hinton agar and discs infused with 10 µL of the extract solutions (1 mg per disc) were mounted on the media surface. Control discs containing fluconazole and rifampicin (10 μg/disc) were used as controls. The sample extracts were incubated at 37 degrees centigrade for 24 h, and the experiments were repeated twice. Thediameters of the zones of inhibition were evaluatedand recorded for antibacterial activity, and inhibition zone diameters greater than 9 mm were evaluated [33].

#### 2.7.2. Determining Minimum Inhibitory Concentration (MIC)

The MICs were calculated using the broth microdilution process. The extracts were examined for solubility in 10% DMSO and then distilled to an appropriate ratio. A sequential ½ dilution of extracts was directly calculated in a microwell plate comprising Mueller microbial broth to achieve a concentration from 0.0125 to 12.8 mg/mL. To offer a final concentration of 5 to 105 CFU/mL in each well, bacterial and fungal inocula were added. The control was used as astandard medication comprising fluconazole and rifampicin at final concentrations from 12 ± 0.11 to 20 ± 0.11 for fluconazole and 24 ± 0.14 to 36 ± 0.14 μg/mL for rifampicin. The plate extract was sprayed with a sterile sealer and incubated at 37 °C for 24 h. The MICs were considered the lowest concentrations of the stem, flower, and leaf extracts, which inhibited microbial growth entirely. The lower the MICs were, the higherthe extract activity [34].

### 2.8. Statistical Analysis

All the datasets were subjected to ANOVA, and the treatment means were analyzed by using Statistics 8.1 at *LSD* < 0.05, whereas the means related to the absorption and wavelength values of ZnO were plotted in graphs using Origin 2021b [35].

## 3. Results

### 3.1. Analysis of Green synthesis of ZnO NPs

#### 3.1.1. Visual and Ultraviolet Spectroscopy (UV-Vis)

**Figure 1:**
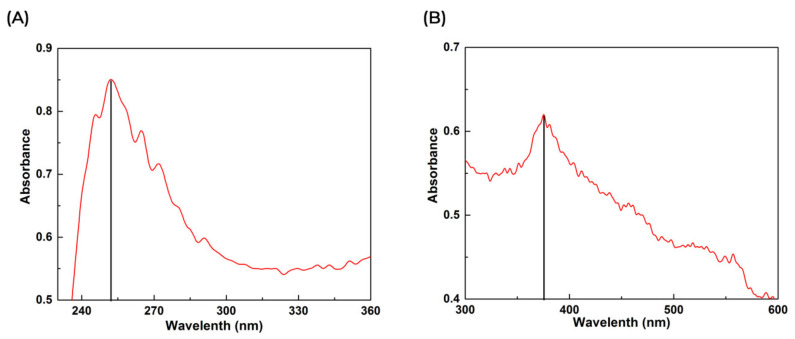
Ultraviolet–visible spectral analysis of synthesized ZnO (**A**) and green synthesized ZnO NPs (**B**).

The synthesized ZnO NPs were analyzed by using UV–Vis spectroscopy. The color deviates to light brown, signifying the formation of ZnO NPs when a 1 M solution of zinc nitrate and aqueous leaf extract of *C. gigantea* was mixed with continuous stirring. The plasma resonance of the ZnO aqueous solution showed a peak of 252.1 nm, and the colloidal solution of ZnO NPs showed a broad peak at 375.6 nm during UV–Vis spectroscopy. This value indicated the absorption spectrum of ZnO NPs (Figure 1A,B).

#### 3.1.2. Scanning Electron Microscopy (SEM)

**Figure 2:**
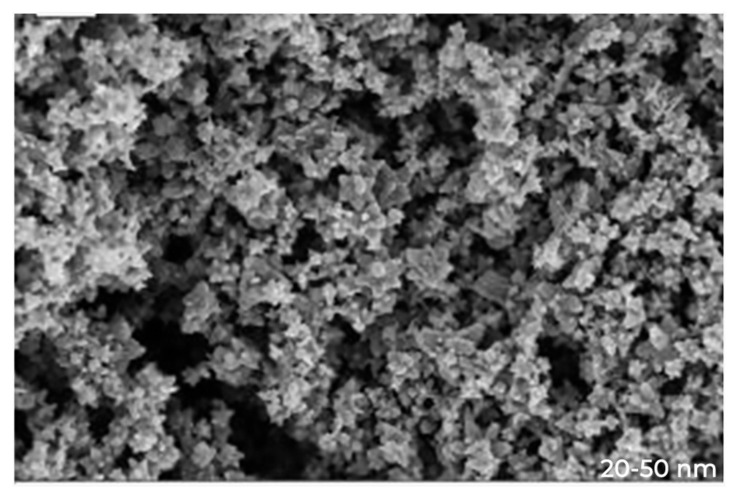
SEM image of ZnO nanoparticles.

The surface morphology of the synthesized ZnO NPs was explored by using SEM. Typical SEM micrographs display many agglomerated particles with irregular spherical morphology with an average size of 80–100 nm. Energy-dispersive X-ray spectra revealed the surface chemical composition ofthe synthesized ZnO NPs (Figure 2).

#### 3.1.3. Energy Dispersive X-ray Analysis (EDX)

**Figure 3:**
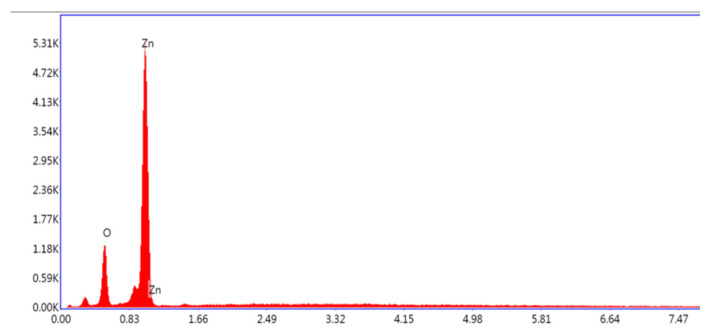
Energy-dispersive X-ray (EDX) spectrum of green-synthesized ZnO NPs.

Analysis of the ZnO nanoparticles by EDX spectrum confirmed the signal characteristic of zinc and oxygen only. All the presented peaks are assigned for Zn and O without any unknown signals, provingthe purity of ZnO nanoparticles by calcination (Figure 3).

#### 3.1.4. FT-IR Spectroscopy

**Figure 4:**
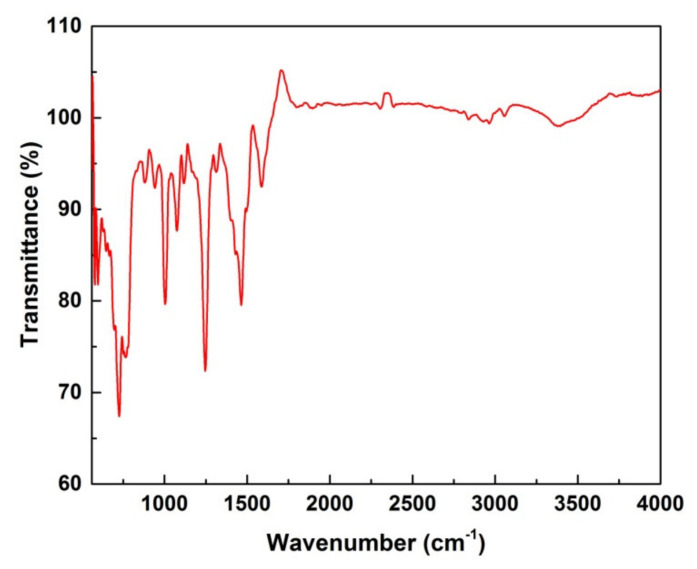
FT−IR analysis of ZnO nanoparticles.

FTIR analysis was performed to determine the functional groups routing the synthesis and stabilization of ZnO NPs. The broad peak 3595.6 cm^−1^ indicated the -OH stretching vibrations. The sharp peak present in the range of 2353.1 cm^−1^ indicated the free carbonyl group. The peaks at 1716.6 cm^−1^ are due to the presence of stretching vibrations of C=O bonds due to non-ionic carboxylic groups and may be assigned to carboxylic acids or their esters in the natural compound. The band at 1340.50 cm^−1^ is due to the presence of the C-O-H bending mode. It is apparent that the intensity of absorption in the region of 617.2 cm^−1^ is characteristic of hexagonal phase Zn-O vibrations at 350 °C. These findings suggested that biological molecules could possibly act as hydrolyzing agents for metal oxide nanoparticle synthesis (Figure 4). 

### 3.2. Comparison of Plant Extracts and Nanoparticles

In the current study, an experiment was carried out to check antimicrobial and enzyme inhibition activities against disease-causing bacteria and fungi. We used plant water, methanol, and acetone extracts from different parts of plants and metal salts (zinc acetate), which are soluble in water. Zinc nanoparticles and plant extracts revealed significant antimicrobial and inhibitory activity against different bacterial and fungal strains.

### 3.3. Determination of AChE and BChE Inhibitory Activity

**Figure 5:**
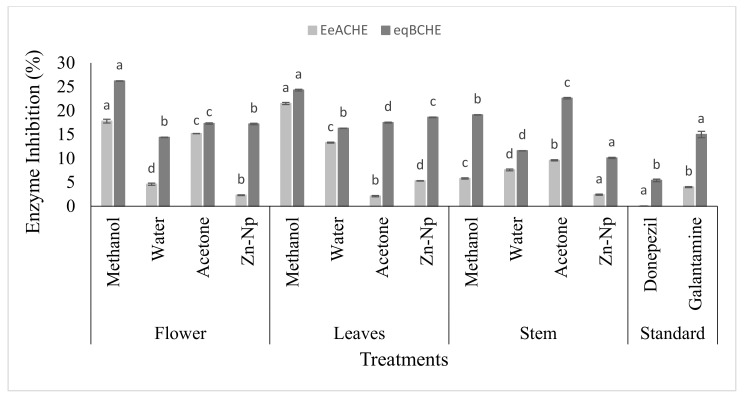
In vitro *AChE* and *BChE* inhibitory activities of *Calotropis gigantea* extracts and zinc nanoparticles. Bars with different letters are significantly different from each other (*p* < 0.05).

The *AChE* and *BChE* inhibitory activities were analyzed at different concentrations of methanol, water, and acetone extracts of *C. gigantea*, used as the tested medicinal plant, and donepezil and galantamine, used as standard drugs. Cholinesterase inhibitors were also observed to show potential. The results showed that all the extracts were inactive compared to drugs such as donepezil for the inhibitory activity of *AChE* and *BChE*. On the other hand, few extracts were inactive compared to the drug-like galantamine forthe inhibition of *AChE* and *BChE*, except the water extract and Zn-Np, which were active for the inhibitory activity of *AChE* and *BChE*. In leaves, the extracts of methanol, acetone, water, and nanoparticles showed IC_50_ values higher than that of the drug (Table 1). All four compounds showed IC_50_ values, and the inhibitory activities of *AChE* and *BChE* were higher than those of the standards. All extracts of *C*. *gigantea* revealed the inhibitory activity of *AChE* and *BChE* against the drug (Figure 5).

### 3.4. Urease Assay

**Figure 6:**
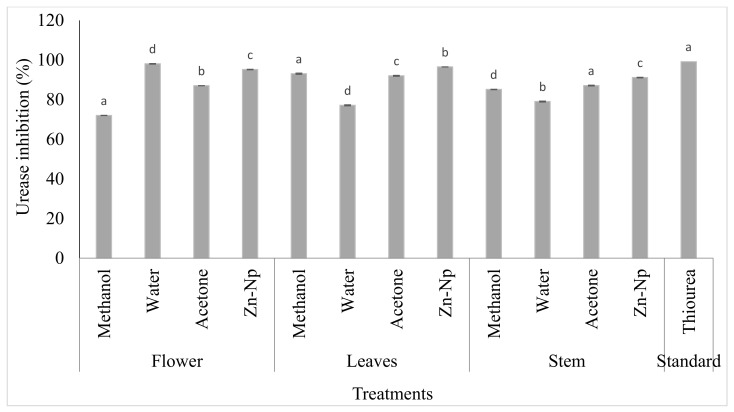
In vitro urease inhibitory activity of *Calotropis gigantea* extracts and zinc nanoparticles. Bars with different letters are significantly different from each other (*p* < 0.05).

These assays were conducted to evaluate the inhibitory activity of zinc nanoparticles and plant extracts. The results showed that the IC_50_ values of water, acetone extracts, and zincnanoparticles were higher (23.14, 25.03, and 22.1 µM, respectively) than thiourea (21.25 µM). The leaf extract of methanol exhibited much higher activity against urease inhibition, with the IC_50_ value being low (17.15 µM). It was observed that zinc nanoparticles and flower extract of acetone showed lower IC_50_ values (17.15 and 16.1 µM) than standard thiourea (21.25 µM). No inhibition of urease in stem extracts of water, acetone, methanol, and zinc nanoparticles had an IC_50_ value higher (31.04, 23.08, and 30.1 µM, respectively) than standard thiourea (17.15 µM). All extracts had no activity against urease, but the methanol extract exhibited much higher activity against urease inhibition (Table 1 and Figure 6).

### 3.5. Antifungal Activity of Zinc Nanoparticles and Plant Extracts

**Figure 7:**
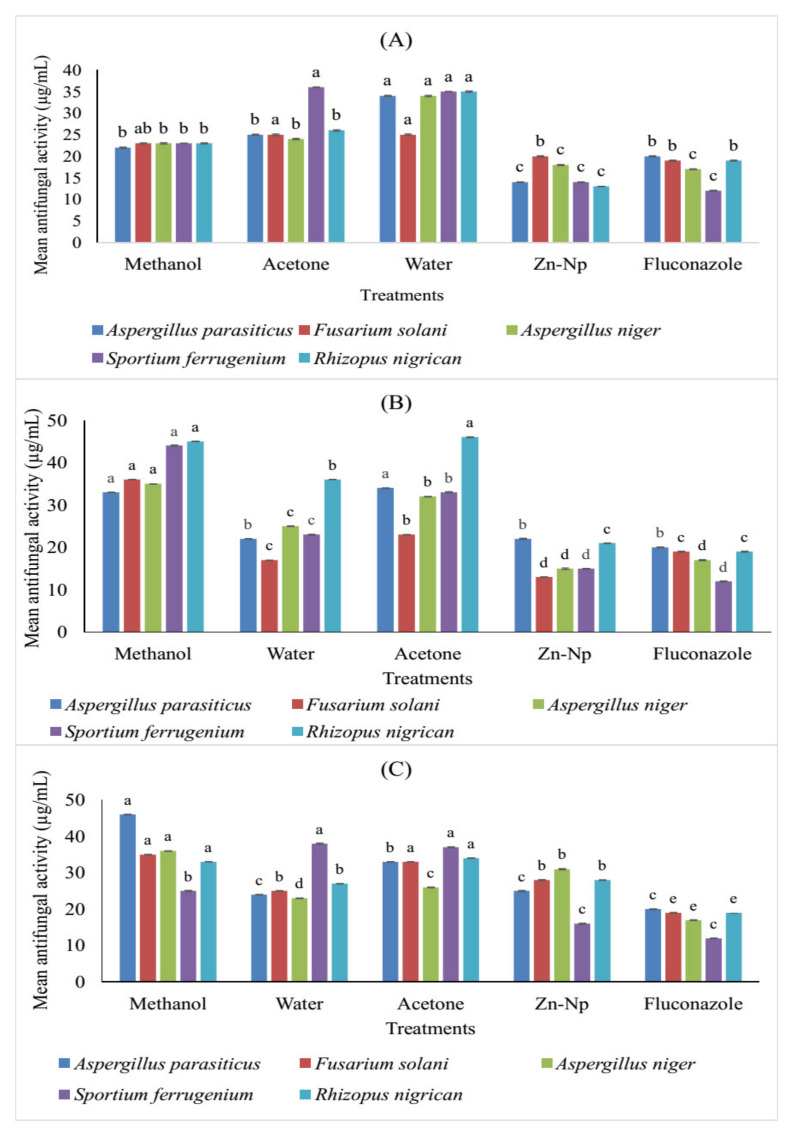
Comparison (MICs) in (µg/mL) different concentrations offlower (**A**), leaf (**B**), and stem (**C**) extracts of *C. gigantea* along with nanoparticles against selected fungal strains. Bars with different letters are significantly different from each other at *p* = *0.05*.

It was observed that zinc nanoparticles exhibited greater antifungal activity against *R. nigricans*, *A. niger*, *F. solani*, and *A. paracistic* than all extracts of flowers, leaves, and stems along with the control. On the other hand, it was observed that leaf extract significantly inhibited the activity of *A. niger*. The results revealed that none of the plant extracts or nanoparticles inhibited the activity of *S. ferrugenium* (Table 1 and Figure 7).

### 3.6. Antimicrobial Activity of Zinc Nanoparticles and Plant Extracts

**Figure 8:**
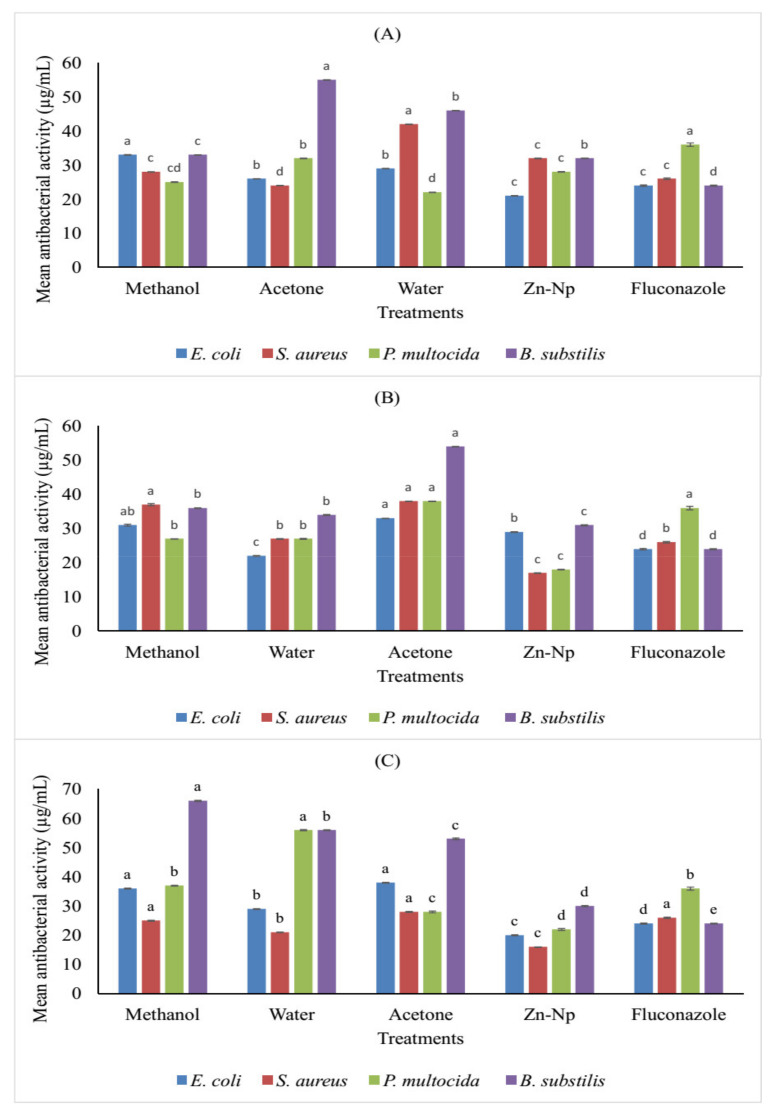
Comparison (MICs) in (µg/mL) different concentrations offlower (**A**), leaf (**B**), and stem (**C**) extracts of *C. gigantea* along with nanoparticles against selected bacterial strains. Bars with different letters are significantly different from each other at *p* = 0.05.

The antimicrobial actions of the plant extracts, nanoparticles, and standard antibiotics were found to have antibacterial activity against the tested microorganisms. The results revealed that antibacterial activity was non-significant in methanol, water, and acetone extracts of flowers against *B. subtilis*, and zinc nanoparticles showed significant activity against *E. coli.* On the other hand, the antibacterial activity was significant in methanol, acetone, zinc nanoparticles, and water extracts against *P. multocida*. In the case of *S. aureus*, the drug was only active in the water extract rather than acetone, zinc nanoparticles, and methanol extracts. Water and methanol extracts of leaves and zinc nanoparticles exhibited antibacterial activity toward *E. coli* and *S. Aureus*, but these extracts and NPs were significant against *P. multocida*. Zinc nanoparticles showed a higher degree of antimicrobial potential against *S. aureus*, *E. coli*, and *P. multocida* than all extracts of the stem. The results showed that *P. multocida* and S. aureus were the most sensitive bacteria to all plant extracts, while *E. coli* is mildly resistant. In contrast, *B. subtilis* was the most resistant microorganism (Table 1 and Figure 8).

## 4. Discussion

The UV-Vis spectrum showed a broad peak at 375.2 nm due to the surface Plasmon absorption of ZnO NP. In agreement with our results, the authors of the reference [36] have reported the absorption peak of ZnO NP at 375 nm, due to surface Plasmon resonance. The authors of reference [37] also reported that the Azadirachta indica-mediated synthesis of ZnO NP was characterized by a maximum absorbance peak at 370 nm. As far as SEM is concerned, most of the nanoparticles synthesized using plant products are spherical in shape [38,39]. Bio-fabricated ZnO NPs using *L. acidissima* were also predominantly mono-dispersed and spherical in shape with an average size of 12–53 nm [40]. Furthermore, ZnO NPs fabricated using the *Aloe vera* were spherical, oval, and hexagonal with an average size ranging from 8–18 nm [36] while the authors of the reference [40] reported spherical and hexagonal ZnO NPs with sizes ranging from 20 to 65 nm in *Lobelia leschenaultiana*. The EDX spectrum of ZnO NPs shows only the peaks of zinc and oxygen elements, which confirm the presence of ZnO NPs. The weak peaks are observed from S and Cl elements along with Zn and O elements due to the X-ray emission from the macromolecules such as alcohol or phenolic compounds [41]. As far as the FT-IR is concerned, the high-intensity band around 440–630 cm^−1^ is due to the stretching mode of the zinc and oxygen bond [42,43]. Further, the absorption bands are located at around 1727 and 1390 cm^−1^, representing C=O stretching vibrations [41]. The phenol molecules are present at 3412 cm^−1^ stretching vibrations, and the 1610 cm^−1^ band reveals the carboxylate group [40]. The band observed at 1633.62 cm^−1^ represents the stretching vibrations of C=O of the amide group [44]. Overall, these peaks indicate that these different functional groups from biomolecules have probably “capped” ZnO to prevent agglomeration, thus stabilizing the medium [45,46,47]. 

The trend regarding the use of natural products has increased, and active plant extracts are often used to discover new drugs [48], synthesize nanoparticles [34], and determine antimicrobial activity [49] in antioxidant and enzyme inhibition studies [50]. The extract of *C. gigantea* leaves, flowers, and stems along with green-synthesized zinc nanoparticles showed significant activity against bacteria and fungi. These extracts were used to determine *AChE*, *BChE*, and urease inhibition activity because of their potential clinical significance on different microbial strains. The results revealed that 6 out of 24 extracts were considered active against *AChE* and *BChE*, including water extracts of stems and flowers, but in the case of leaves, acetone extract was also active. All these extracts showed significant inhibitory activities and have more potential forthe inhibition of *BChE* than *AChE*. Among all the extracts, one of the most active extractscompared to the standard (15.0 ± 0.67) was the flower extract for the inhibition of *BChE* (14.4 ± 0.06). The inhibitory activity of these parts of the plant against these enzymes is due to the presence of alkaloids such as terpenoids, glycosides, and coumarins. These alkaloids make it a good inhibitor of the enzyme [51]. Zinc nanoparticles and water extract of the stem were active for inhibition of *AChE* and *BChE*. Zinc extract has less potential for the inhibitory activity of acetylcholinesterase and butyrylcholinesterase, which includes transferase and is considered a source of the development of acetyl-CoA. Acetyl-CoA is the neurotransmitter. Cholinesterase enzymes disconnect acetyl-CoA and then block the transmitted signal through acetyl-CoA. Enzymatic interaction with nanoparticles will inhibit the enzyme and thus promote cholinesterase degradation [52]. It was observed that three compounds exhibited much higher activity against urease inhibition: methanol extract of leaves, whose IC_50_ value was low (17.15 µM), and acetone extract of flower (19.03 ± 0.06) compared to the control drug thiourea (21.25 µM). Zinc nanoparticles and extract of the flower were active (16.1 µM) compared to the control drug thiourea (21.25 µM). Urease inhibition with zinc nanoparticles was due to its highly probable contact with the active site of urease having a sulfuryl group, resulting in a decrease in catalytic properties. It influenced the activity of enzymes by modifying the enzyme-substrate complex, such as alterations of the protein and metal ligand-binding structure at active sites [53]. The best among all extracts was the acetone extract of flower for inhibition of theurease enzyme due to several molecules that have been identified as urease inhibitors by researchers, such as phosphor amides, heterocyclic compounds, quinones, heavy metal ions, and thioles. In addition, 1,2-Benziso-3(2H)-one derivatives are also a major family of new classes of urease inhibitors [54]. Some natural flavonoids that are available on a commercial scale have shown urease inhibitory activity [55]. Zinc nanoparticles exhibited antifungal activity against *A. paracistic*, *F. solani*, *A. niger*, and *R. nigarican* in flower and leaf extracts, while the water extract of leaves was active against *F. solani* compared to other extracts, such as methanol and acetone. *Fusarium solani* was the most sensitive fungus, and *A. paracistic*, *A.niger*, *S. ferrugenium*, and *R. nigrican* were the most resistant microorganisms. Zinc nanoparticles of leaves showed activity due to molecular mechanisms for antiviral activity of NPs, including nanoparticle size, surface load, or chemistry, and are accused of including cell membrane modifications, failure of respiratory function, oxidative damage of lipids, production of ROS, DNA untangling, protein thiol, nitrosation process, or metabolic pathway degradation. The water extract of leaves showed activity due to its antifungal action evaluated from the past, as the species of *F. solani* produces several secondary metabolites commonly referred to as mycotoxins. Secondary metabolites are known to be more resistant to strong carcinogenic agents, hepatotoxins, teratogenic agents, and immunosuppressants, making them resistant to drugs used either in plant extracts to produce antifungal action in plants [56]. In the present study, it was reported that nanoparticles and plant extracts of flower extract, zinc nanoparticles, and water extracts were active against *E. coli* compared to standards. In comparison with methanol and acetone extracts against microbes, water extracts and zinc nanoparticles showed more activity. Zinc nanoparticles showed more activity in contradiction of *E. coli*, *S. aureus*, and *P. multocida* because metal nanoparticles such as zinc associate with the sulfhydryl group in the microbial wall, interfering with bacterial cell membranes, function of the enzymes, respiratory chains, and the proliferation of the cells and killing them [57]. On the other hand, the water extract showed a maximum zone of inhibition against *E. coli* and *S. aureus* due to bioactive compounds in *C. gigantea*. These bioactive compounds (saponins, tannins, flavonoids, and alkaloids) are effective against different kinds of microbes [58], while tannins and proline-rich proteins form irreversible complexes that lead to cell-protein inhibition [58,59,60,61].

## 5. Conclusions

The findings of the present research demonstrated that the antimicrobial activity and inhibition of the enzymes varied with the different components of the plant species. Medicinal plants, including water extracts of the flowers, stems, and leaves of *C. giganteash*, could be used as bioreduction toolsfor the development of Zn-NPs. Zinc nanoparticles reduced the growth of *E. coli*, *S. aureus*, and *P. multocida* bacterial and fungal species *A. parasiticus*, *F. solani*, *A. niger*, and *R. nigarican*. Water extracts of *C. gigantea* have a greater ability to reduce pest growth than methanol and acetone extracts. Zinc nanoparticles can inhibit cholinesterase activity more remarkably than urease inhibition activity.Further studies are needed to evaluate the biological activities of the bioactive compounds present in plant extracts.

## Figures and Tables

**Table 1:** *AChE*, *BChE* and increase inhibitory activities IC_50_ (μM ±SEM)A** of (*Calotropis gigantea*) extracts and nanoparticles.

Plant Parts	Solvents	EeAChE and eqBChE SI *	IC50 µM
Flower	Methanol	1.3	49.03 ± 0.08
	Acetone	1.2	^b^ 19.03 ± 0.06
	ZnNPs	1.3	16.1 ± 0.15
	Water	3.9	30.07 ± 0.18
Leaves	Methanol	1.2	43.06 ± 0.16
	Acetone	8.6	25.03 ± 0.09
	ZnNPs	1.4	22.1 ± 0.03
	Water	1.3	23.14 ± 0.26
Stem	Methanol	3.3	^b^ 17.15 ± 0.07
	Acetone	3.8	23.08 ± 0.11
	ZnNPs	1.7	30.1 ± 0.13
	Water	3.8	31.04 ± 0.17
A**	Donepezil	180	-
	Galantamine	3.7	-
	Thiourea	-	21.25 ± 0.17

* Selectivity Index = (*BChE*/*AChE*) IC_50_ ratio. For highly active compounds represented by bold values; A** = Value is represented as a mean (standard mean error of at least three tests, and ^b^ bold values represent highly active compounds.

## Data Availability

Not applicable.
